# Effect of cinnamaldehyde on Cav-1 and Survivin expression in epilepsy: A protocol of systematic review and meta-analysis

**DOI:** 10.1097/MD.0000000000020459

**Published:** 2020-06-05

**Authors:** Jia-Nan Yu, Cai-Fang Yue, Ke-Jian Wang, Nan-Nan Chi, Xin Li

**Affiliations:** a Third Ward of Neurology Department; b Department of Critical Medicine; c Fourth Ward of Neurology Department; d Second Ward of Gastroenterology Department; e First Ward of Neurology Department, First Affiliated Hospital of Jiamusi University, Jiamusi, China.

**Keywords:** Cav-1, cinnamaldehyde, effect, epilepsy, survivin

## Abstract

**Background:**

This systematic review aims to assess the effect of cinnamaldehyde on Cav-1 and Survivin expression in epilepsy.

**Methods:**

We will search Cochrane Library, PUBMED, EMBASE, CINAHL, Web of Science, Google Scholar, PsycINFO, WANGFANG, VIP, CBM, and CNKI from their inceptions to the March 31, 2020, without language restrictions. Two authors will independently carry out searching literature records, scanning titles and abstracts, full texts, collecting data, and assessing risk of bias. RevMan 5.3 software will be used for statistical analysis.

**Results:**

This systematic review will investigate whether cinnamaldehyde is effective on Cav-1 and Survivin expression in epilepsy.

**Conclusion:**

Its findings will provide helpful evidence for the effect of cinnamaldehyde on Cav-1 and Survivin expression in epilepsy.

Systematic review registration: INPLASY202040152.

## 1 Introduction

Epilepsy is one of the most common chronic neurological diseases,^[1–4]^ which is characterized by an enduring predisposition to generate seizures.^[5]^ It can affect people of any ages, irrespective their races, economic status, educational background, social class, and geographical locations.^[6–11]^ Many factors can provoke and induce this condition, including neurobiologic, cognitive, psychological, and social consequences.^[12–15]^ Although lots of treatments are available for seizures, its efficacy is limited.^[16–19]^ Thus, it is still very important to explore more effective medications for this disorder.

Previous studies have found that Cav-1 and Survivin has association with epilepsy,^[20–25]^ and several studies have examined cinnamaldehyde the affect Cav-1 and Survivin expression in epilepsy,^[25,26]^ which can help find out new potential medications for epilepsy. However, all their conclusions are based on the single study and no study has been conducted to address this topic comprehensively and systematically. Thus, this study will explore the effect of cinnamaldehyde on Cav-1 and Survivin expression in epilepsy.

## 2 Methods

### 2.1 Study registration

This study was registered and funded on INPLASY202040152. It has been reported based on the guidelines of the Preferred Reporting Items for Systematic Reviews and Meta-Analysis Protocol statement.^[27,28]^

### 2.2 Criteria for considering studies for this review

#### 2.2.1 Types of studies

This study will only consider case-controlled studies (CCSs) of cinnamaldehyde on Cav-1 and Survivin expression in epilepsy. However, studies of nonclinical studies and noncontrolled trials will be excluded in this study.

#### 2.2.2 Types of subjects

This systematic review will include subjects who were diagnosed as epilepsy.

#### 2.2.3 Types of exposures

In the experimental group, all epilepsy subjects received cinnamaldehyde in this study.

In the control group, all epilepsy subjects did not receive any treatment in this study.

#### 2.2.4 Types of outcome measurements

Primary outcomes are gene and protein expressions of Cav-1 and Survivin. Gene expression was measured by real-time quantitative real-time polymerase chain reaction. Protein expression was detected by immunofluorescence or western blot test.

Secondary outcomes are patch-clamp whole-cell mode voltage clamp recording, and survivin apoptosis factor, as measured by flow cytometry.

### 2.3 Search methods for identification of studies

#### 2.3.1 Electronic databases

We will carry out comprehensively search from Cochrane Library, PUBMED, EMBASE, CINAHL, Web of Science, Google Scholar, PsycINFO, WANGFANG, VIP, CBM, and CNKI. All these electronic databases will be searched from their inceptions to the March 31, 2020, without language and publication status restrictions. We will present a detailed search strategy for Cochrane Library in Table 1. In addition, we will adapt similar detailed search strategy to the other electronic databases.

**Table 1:**
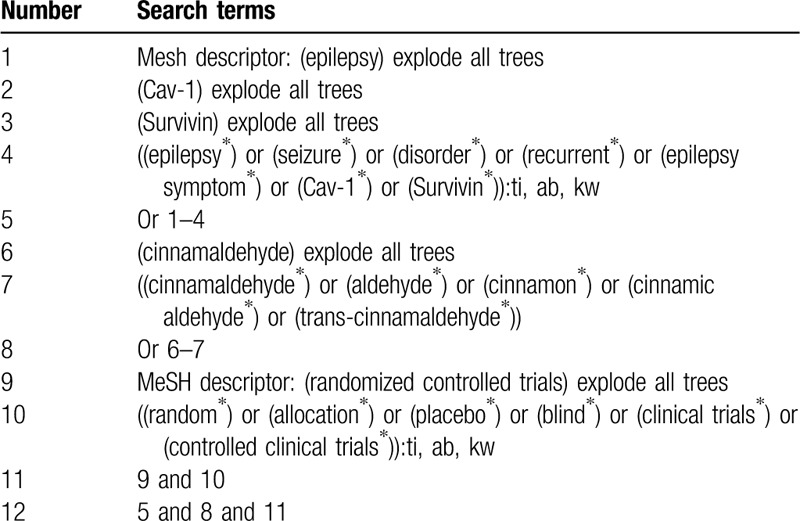
Search strategy for Cochrane Library.

#### 2.3.2 Searching other resources

This study will also search ongoing studies, clinical registry, and reference lists of relevant studies.

### 2.4 Data collection and analysis

#### 2.4.1 Study selection

We will carry out study selection according to the pre-designed eligibility criteria. Two authors will independently screen the titles and abstracts of all literature records. We will exclude all irrelevant studies, and full-text of all remaining studies will be further identified. If any different opinions occur between 2 authors, we will invite a third author to solve it via discussion. The whole process of study selection is demonstrated in the flowchart.

#### 2.4.2 Data collection and management

We will utilize a previous designed data collection form to extract the data. Two independent authors will conduct data collection, and any divergences between 2 authors will be solved by a third author though discussion. The following information will be extracted: study information, such as title, time of publication, first author, and so on; patient characteristics, such as race, age, and so on; study methods, such as sample size, randomization, blind, concealment, and so on; intervention details, such as dose, duration, frequency, and so on; and outcomes, such as primary and secondary outcomes, and safety.

#### 2.4.3 Missing data dealing with

If there is unclear or insufficient or missing information, we will contact primary authors to request it. If we cannot get any reply, we will pursue analyses based on the available data.

#### 2.4.4 Assessment of risk of bias of included studies

Two authors will independently conduct the risk of bias for each eligible study using Cochrane risk of bias. It has 7 domains, and each field is further assigned as low, unclear, and high risk of bias. Any disagreements between the 2 authors will be solved by a third author through discussion. We will summarize the results of risk of bias assessments in Risk of Bias Table.

#### 2.4.5 Measurement of treatment effect

For dichotomous values, we will calculate them as risk ratio and 95% confidence intervals. For continuous values, we will calculate them as mean difference or standardized mean difference and 95% confidence intervals.

#### 2.4.6 Assessment of heterogeneity

We will use *I*^2^ statistics to check the heterogeneity among eligible studies. The value of *I*^2^ ≤ 50% means low heterogeneity; and the value of *I*^2^ > 50% indicates substantial heterogeneity.

#### 2.4.7 Data synthesis

We will apply RevMan 5.3 software for statistical analysis in this study. A meta-analysis will be conducted if low heterogeneity exists among included studies on the same interventions and outcomes. A fixed-effects model will be utilized if the heterogeneity is low. On the contrary, a random-effect model will be employed if the heterogeneity is significant. Then, subgroup analysis and meta-regression test will be performed to explore sources of substantial heterogeneity.

#### 2.4.8 Publication bias

We will carry out Funnel plot and Egger regression test to check if there is any publication bias when more than 10 studies are included.^[29]^

#### 2.4.9 Subgroup analysis

We will undertake subgroup analysis based on the different interventions, controls, and outcome tools.

#### 2.4.10 Sensitivity analysis

We will exclude studies with a high risk of bias to identify the robustness and stability of pooled outcomes.

### 2.5 Dissemination and ethics

No ethical approval is needed, because this is a literature-based study. We are expected to publish this study at peer-reviewed journals.

## 3 Discussion

Several studies have reported the effect of cinnamaldehyde on Cav-1 and Survivin expression in epilepsy. However, no systematic review with sufficient evidence has investigated this issue. Therefore, this study will systematically appraise the effect of cinnamaldehyde on Cav-1 and Survivin expression in epilepsy. The findings of this study may fulfill the gap in this field and may provide evidence to further explore potential medicine for epilepsy, which may benefit both future research and clinical practice.

## Author contributions

**Conceptualization:** Jia-nan Yu, Cai-fang Yue, Ke-jian Wang, Nan-nan Chi, Xin Li.

**Data curation:** Jia-nan Yu, Cai-fang Yue, Xin Li.

**Formal analysis:** Jia-nan Yu, Cai-fang Yue, Ke-jian Wang, Nan-nan Chi, Xin Li.

**Investigation:** Xin Li.

**Methodology:** Jia-nan Yu, Cai-fang Yue, Nan-nan Chi.

**Project administration:** Xin Li.

**Resources:** Jia-nan Yu, Cai-fang Yue, Ke-jian Wang, Nan-nan Chi.

**Software:** Jia-nan Yu, Cai-fang Yue, Ke-jian Wang, Nan-nan Chi.

**Supervision:** Xin Li.

**Validation:** Jia-nan Yu, Cai-fang Yue, Ke-jian Wang, Nan-nan Chi, Xin Li.

**Visualization:** Jia-nan Yu, Cai-fang Yue, Xin Li.

**Writing – original draft:** Jia-nan Yu, Cai-fang Yue, Ke-jian Wang, Nan-nan Chi, Xin Li.

**Writing – review & editing:** Jia-nan Yu, Ke-jian Wang, Nan-nan Chi, Xin Li.
